# Sequential Analysis of Murine Myelofibrosis Models Using a Novel Deep Learning‐Based Fibrosis Quantitative Method

**DOI:** 10.1002/jha2.70273

**Published:** 2026-04-24

**Authors:** Toshikuni Kawamura, Takaaki Maekawa, Keita Kouzu, Hiraku Ogata, Noriaki Tachi, Shoichiro Kato, Yukiko Osawa, Takahiro Einama, Kimiya Sato, Shinichi Kobayashi

**Affiliations:** ^1^ Division of Hematology Department of Internal Medicine National Defense Medical College Tokorozawa Saitama Japan; ^2^ Division of Palliative Care National Defense Medical College Hospital Tokorozawa Saitama Japan; ^3^ Department of Surgery National Defense Medical College Tokorozawa Saitama Japan; ^4^ Department of Basic Pathology National Defense Medical College Tokorozawa Saitama Japan

**Keywords:** deep learning, extramedullary hematopoiesis, hematopoietic stem cell, *JAK2*V617F mutation, primary myelofibrosis, reticular fibers, splenomegaly

## Abstract

**Introduction:**

Primary myelofibrosis (MF) is characterized by MF, splenomegaly, and extramedullary hematopoiesis. MF initially presents with reticular fibers (RFs) and progresses to increased collagen fiber deposition in the advanced stages. Although recent clinical trials have adopted MF improvement as an evaluation criterion, current diagnostic methods rely primarily on qualitative assessments based on the presence or absence of reticular and collagen fibers. Therefore, detecting subtle changes during the early stages of MF can be challenging.

**Methods:**

We developed a novel deep learning‐based method for quantitatively evaluating MF by measuring RFs as an indicator. Unlike collagen fibers, RFs are detectable from the early stages of MF and increase as the disease progresses. Moreover, based on the hypothesis that splenic fibrosis progresses in parallel with MF, we applied this method to evaluate RFs in the spleen. Using these methods, we analyzed temporal changes in fibrosis, splenomegaly, and hematopoietic stem cell dynamics over time in two MF models: a drug‐induced fibrosis model using romiplostim and a *Jak2*V617F gene–transformed mouse. Additionally, we examined correlations between our quantitative fibrosis measurements and clinical data, including MF grade and genetic mutations.

**Results:**

Our findings revealed that in *Jak2*V617F gene–transformed mice, splenomegaly and extramedullary hematopoiesis in the spleen occurred earlier than MF. Furthermore, the quantitative fibrosis method significantly correlated with MF grade in patients with myeloproliferative neoplasms and the *JAK2*V617F mutant allele burden.

**Conclusion:**

Our novel deep learning‐based method successfully captured temporal changes in bone marrow and spleen fibrosis and shows potential for clinical application.

**Trial Registration:**

The authors have confirmed clinical trial registration is not needed for this submission

## 1 Introduction

Primary myelofibrosis (PMF) is a myeloproliferative neoplasm (MPN) characterized by the progressive fibrosis of the bone marrow (BM), extramedullary hematopoiesis (EMH), splenomegaly, and leukoerythroblastosis. Most patients with PMF harbor driver mutations, such as *JAK2*, *CALR*, and *MPL* mutations [1]. These mutations activate the thrombopoietin (TPO) signaling pathway, which plays a crucial role in the proliferation and differentiation of PMF clones [1].

BM fibrosis initially presents with reticular fibers (RFs) and progresses to increased collagen fiber deposition in the advanced stages, which results from PMF clones [1]. Evaluation of BM biopsies involves scoring according to the World Health Organization (WHO) classification, which includes four escalating grades of severity: Grades 0–3 [1, 2]. BM fibrosis (≥ Grade 2) has been incorporated into recent scoring systems, such as the Mutation‐enhanced International Prognostic Scoring System 70 and 70‐plus (MIPSS70 and MIPSS70‐plus) [3]. The primary endpoints of a clinical trial for ruxolitinib, the first‐in‐class JAK2 inhibitor for myelofibrosis (MF), did not include improvement in BM fibrosis but rather focused on the reduction in splenomegaly and general symptoms [4, 5]. However, in the 5‐year final analysis of the ruxolitinib clinical trial, alleviation of BM fibrosis was observed in some patients [6]. Recent research has shown that improvement in BM fibrosis is indicative of disease modification, and changes in BM fibrosis or its reversal have been considered endpoints in clinical trials [7, 8, 9, 10]. However, negative clinical data have been obtained regarding the association between treatment‐induced changes in BM fibrosis and efficacy outcomes such as survival, symptom alleviation, spleen size reduction, and anemia response, indicating that the role of BM fibrosis in the context of JAK inhibitor therapy remains unclear [11].

Two issues persist in the evaluation of BM fibrosis: (1) the BM sample is too small to accurately assess the degree of BM fibrosis in general, and (2) the WHO classification only estimates qualitative changes in RFs and collagen fibers and does not reflect the degree of BM fibrosis in a linear fashion [2]. Therefore, a quantitative analysis is needed to reveal the relationship between BM fibrosis and other clinical parameters and compare the effectiveness of newly developed drugs. Two previous studies used an image analysis software to quantify the BM fibrotic area; however, this approach was limited by the need to stain samples simultaneously and under identical conditions [12, 13]. In the present study, we developed a novel deep learning‐based method for quantifying BM fibrosis to overcome this limitation. In this method, we quantified RFs as an indicator of BM fibrosis. Unlike collagen fibers, which emerge only in advanced stages, RFs are detectable in the early stages of BM fibrosis and increase progressively as the disease advances [2]. Furthermore, we applied this method to evaluate spleen fibrosis, which had not been attempted before. RFs, known as Type III collagen fibers, are found primarily in tissues that contain Type I collagen, for example, lung, liver, spleen, and kidney [14]. In addition, spleen and liver stiffness have been reported to be associated with BM fibrosis in patients with MPN [15], which may be related to RF hyperplasia in the spleen. Based on the hypothesis that spleen fibrosis progresses with progress in BM fibrosis, we defined the fibrosis area in our study as the one including the RFs that originally existed in the spleen.

To evaluate this novel deep learning‐based method, we tracked the progression of BM fibrosis over time and analyzed the temporal relationships between PMF‐related events (splenomegaly, BM fibrosis, and spleen fibrosis) and hematopoietic stem cell (HSC) dynamics in two murine models of MF: romiplostim (Rom)‐induced MF mice and *Jak2*V617F transgenic (TG) mice [16, 17]. Furthermore, we applied this assessment to human BM samples and analyzed the relationship to the *JAK2*V617F allele burden assessed using genomic DNA isolated from the peripheral blood (PB) of patients.

## 2 Materials and Methods

### 2.1 Murine MF Models

C57BL/6J mice were purchased from CLEA Japan, Inc. (Tokyo, Japan). Ten‐week‐old female mice were subcutaneously injected with 1 mg/kg Rom on Days 1 and 8 (Rom MF model). Four mice were sacrificed on each of the following Days: 3, 5, 7, 9, 11, and 13. The 10‐week‐old control mice, which were not administered Rom, were sacrificed on Day 1. PB was drawn from the inferior vena cava, and the spleen and femur were extracted. Spleen cells were isolated following the method described in a previous study [18].

The *Jak2*V617F TG mice (Line 2) were kindly provided by Dr. Kazuya Shimoda, Miyazaki University, Miyazaki, Japan [17]. The PB, spleens, and femurs of male and female *Jak2*V617F TG mice were processed as previously described [16]. To account for variations in spleen weights attributable to differences in body weight, spleen weights were expressed as a percentage of body weight in two murine models. For the Rom MF model mice, splenomegaly was defined as a significant increase in the mean percentage of spleen weight relative to body weight compared with control mice, whereas for the *Jak2*V617F TG mice, splenomegaly was defined as a significant increase in the mean percentage of spleen weight relative to body weight compared with 12‐week‐old wild‐type mice of the same strain.

All mice were housed under specific pathogen‐free conditions with temperatures ranging from 20°C to 26°C, humidity levels between 40% and 60%, and a 12‐h light/12‐h dark cycle and had free access to water and food. All experiments were conducted under inhalation anesthesia using isoflurane (Wako Pure Chemical Industries, Osaka, Japan). All mice were euthanized via cervical dislocation at the end of PB collection.

### 2.2 Deep Learning‐Based Fibrosis Quantification

Murine femur specimens were fixed in formalin, demineralized in Morse solution (10% sodium citrate and 22.5% formic acid), embedded in paraffin, and sectioned for silver staining according to the standard protocol [19]. Moreover, murine spleen specimens were fixed in formalin, embedded in paraffin, and sectioned for silver staining according to the standard protocol [19]. All silver staining in this study was conducted by a dedicated technician from the Department of Basic Pathology at the National Defense Medical College. Silver‐stained slides of murine femurs and spleens were digitized using NanoZoomer SQ (Hamamatsu Photonics K.K., Shizuoka, Japan) and uploaded to HALO AI (Indica Labs, Corrales, NM, USA). In the two murine models we used, Masson trichrome staining could not confirm the appearance of collagen fibers at any time point, so we focused our evaluation on RFs as depicted by silver staining.

**FIGURE 1:**
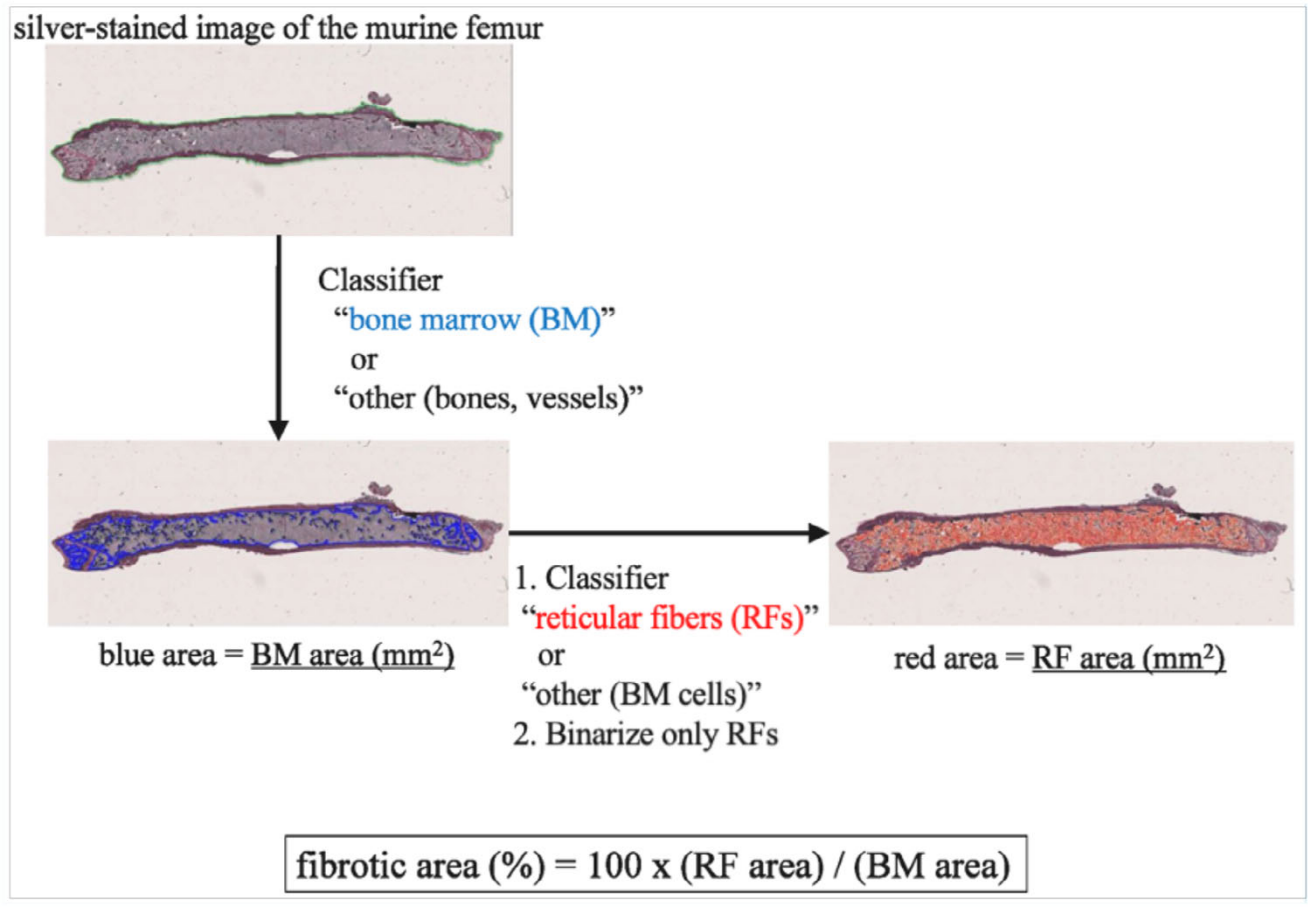
Quantification of BM fibrosis using a deep learning‐based method. The entire murine femur in the digitized slide was semiautomatically annotated (green line). The areas for the BM and RF were measured using two classifiers and the Area Quantification v2 (HALO application). Finally, the fibrotic area was calculated using the following formula: fibrotic area (%) = 100 × (RF area) / (BM area). All sample images were of silver‐stained specimens from *Jak2*V617F transgenic mice. BM, bone marrow; RF, reticulin fiber.

**FIGURE 2:**
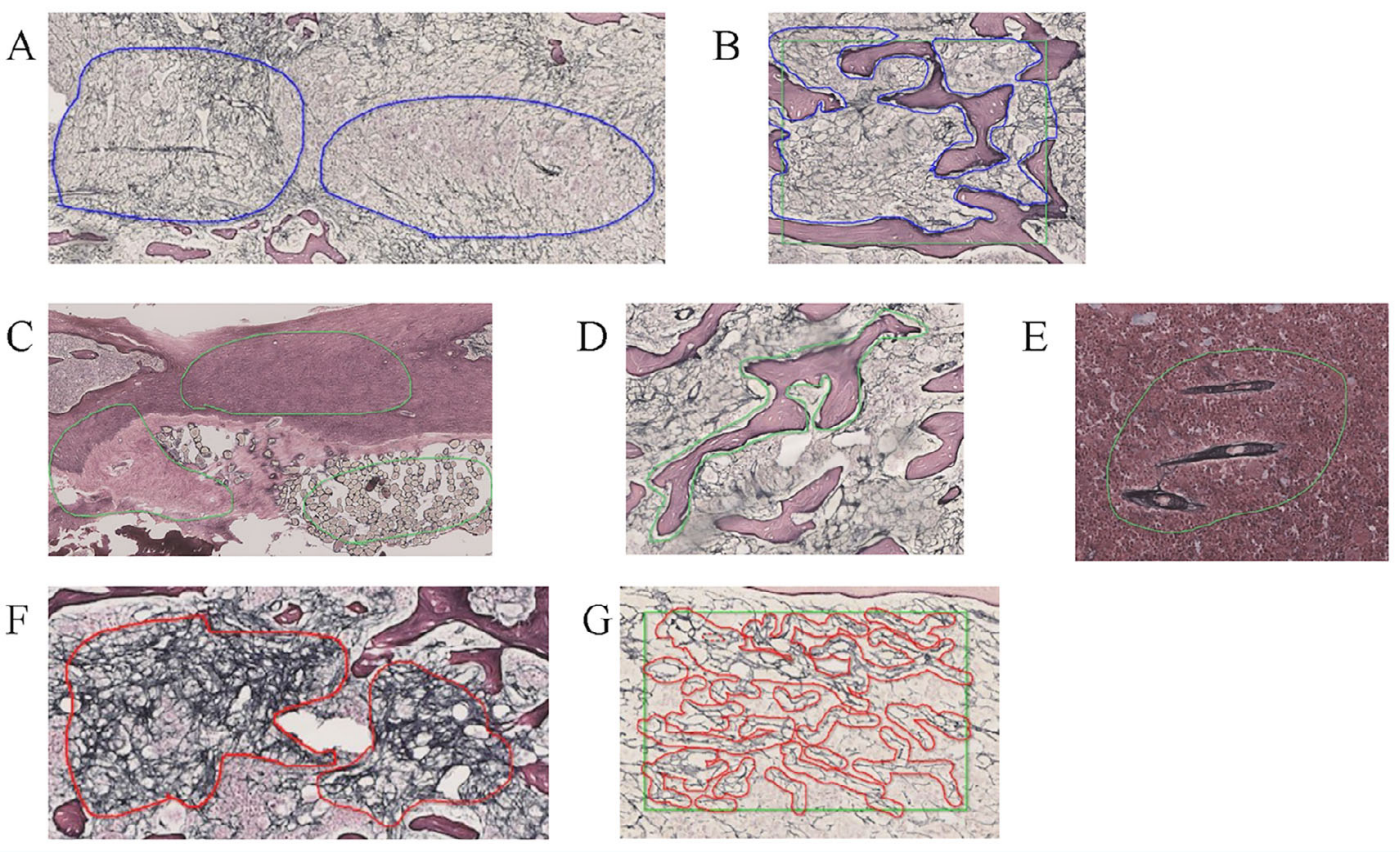
Example images from the training dataset. (A) BM area (blue line). (B) Any region enclosed by the other area (green line) was annotated as the BM area (blue line). (C–E) The other area, which included (C) the bone cortex and connective tissues, (D) bone, and (E) blood vessels (green line). (F) Area containing RFs (red line). (G) Any region was enclosed like the other area (green line), and the RFs within it were marked as the RF area (red line). BM, bone marrow; RFs, reticular fibers.

**FIGURE 3:**
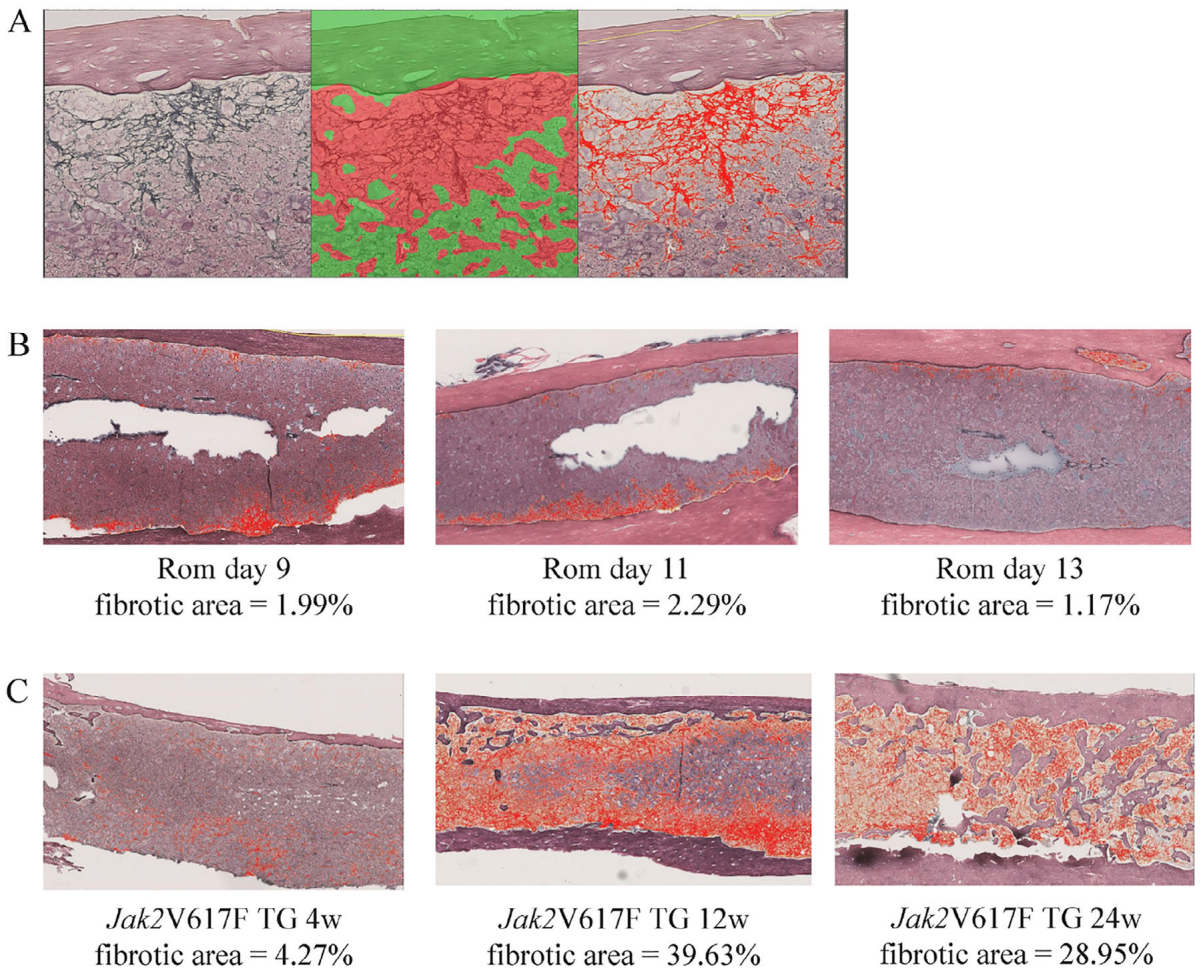
Example images showing the quantification of BM fibrosis. (A) Three cameras show the same field of view. An image of a silver‐stained murine femur (left). Red and green colors indicate the RF area and other area, respectively (middle). RFs in the RF area were binarized (right), and only RFs are represented in red. (B) In the Rom MF model, the percentage of BM fibrotic area was measured on Days 9, 11, and 13 after Rom administration. (C) In Jak2V617F TG mice, the percentage of BM fibrotic area was measured at 4, 12, and 24 weeks of age. BM, bone marrow; MF, myelofibrosis; RFs, reticular fibers; Rom, romiplostim; TG, transgenic.

To quantify fibrosis, the areas of RF in the BM and spleen of mice were measured using HALO AI. Figure 1 shows the sequence of this method. In the classifier—a program that classifies data into specific categories based on a specific algorithm—developed to recognize BM, we annotated the fields of view to include BM and other tissue types, such as bones, connective tissues, and blood vessels (Figure 2A–E), in five randomly selected slides from murine MF models. The annotations were used to train DenseNet v2 for approximately 2 h. The area containing BM of the field of view that was distinct from the other tissue types was considered the BM area (mm^2^). To identify the RF area within the BM region specified earlier, we used deep learning techniques to locate the area containing RFs. In this region, we extracted only the RFs by binarizing the RFs and the background and then calculated the area of the RFs. In the classifier designed to recognize RFs, we annotated the fields of view containing RFs and normal BM in five randomly selected slides from murine MF models (Figure 2F,G). The annotations were used to train DenseNet v2 for approximately 2 h. In the field of view containing RFs, only the RFs were binarized, and the area of the RFs (mm^2^) was measured using Area Quantification v2 (HALO application). In the current analysis, the process of identifying the fields of view containing RFs and binarizing within those regions can be performed in a single step. The fibrotic area (%) was calculated as the percentage of RF area relative to the BM area. Figure 3A shows an example of the results of analysis. Although the areas containing RFs also included normal BM (Figure 3A middle), binarization allowed for the exclusion of normal BM to determine the RF area alone (Figure 3A right).

**FIGURE 4:**
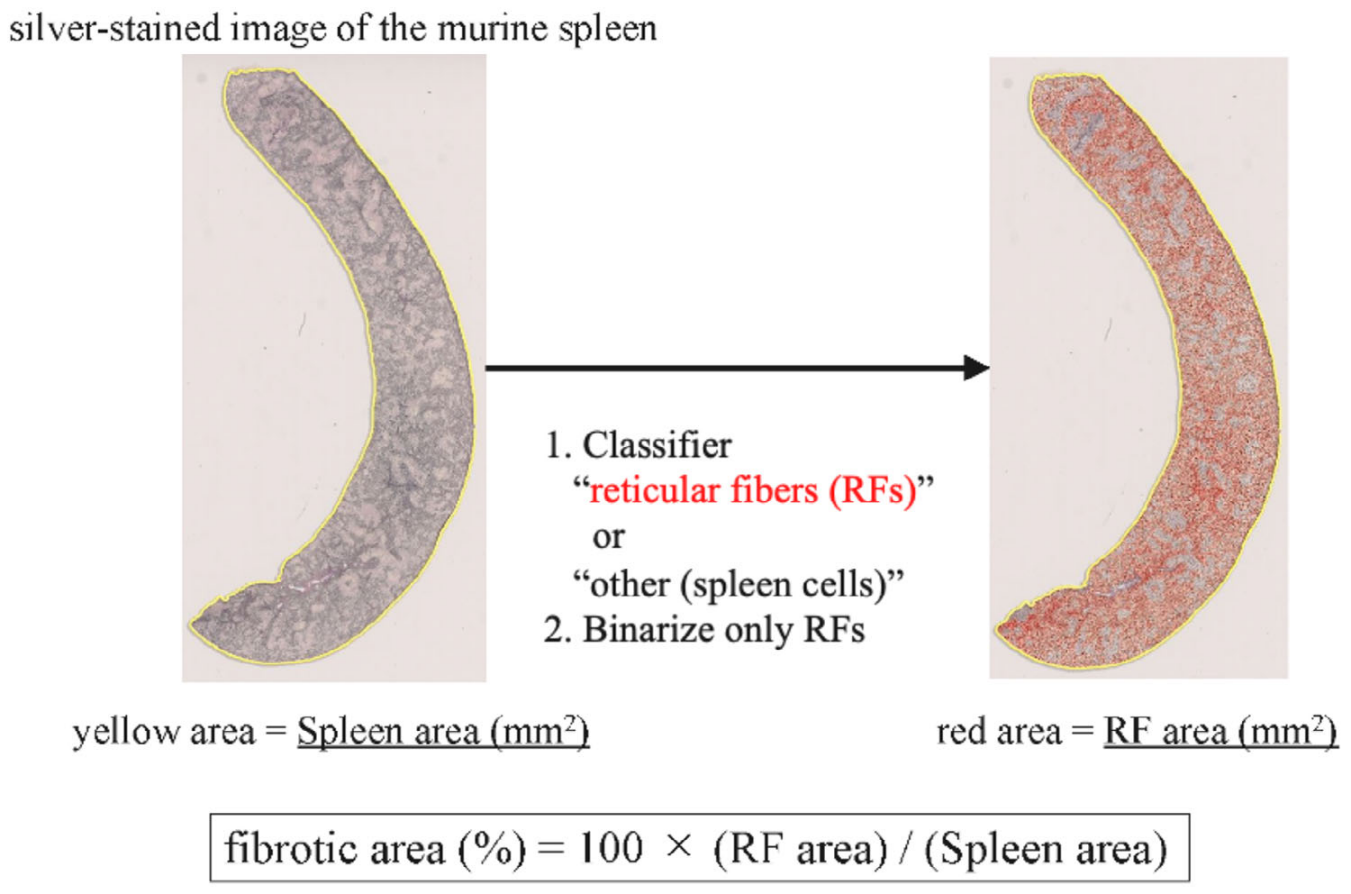
Quantification of spleen fibrosis using a deep learning‐based method. The entire murine spleen on the digitized slide was semiautomatically annotated using a yellow line, and the area within the annotation was defined as the spleen area. The area of the RF was measured using a classifier and Area Quantification v2 (HALO application). Finally, the fibrotic area was calculated using the following formula: fibrotic area (%) = 100 × (RF area) / (spleen area). All sample images were of silver‐stained specimens from Jak2V617F transgenic mice. RF, reticulin fiber.

Figure 4 shows the spleen fibrosis quantification method. The spleen margin was automatically delineated using HALO AI (Figure 4, yellow line), and the area marked in yellow was defined as the spleen area (mm^2^). In the spleen, regions containing RFs and those containing spleen cells and blood vessels were identified, and only the RFs were binarized to calculate their area (mm^2^). The fibrotic area (%) was calculated as the percentage of RF area relative to the total spleen area. BM and spleen fibrosis were assessed in the murine experiments using this method.

### 2.3 Assessment of HSCs in PB and Spleen

Murine PB cells were subjected to hemolysis using BD Pharm Lyse (BD Biosciences, San Jose, CA, USA) according to the manufacturer's instructions. The PB and spleen cells were resuspended in 500 µL of fluorescence‐activated cell sorter (FACS) buffer (phosphate‐buffered saline containing 3% fetal bovine serum) and blocked with 1 µL of Fc block (BD Biosciences) for 10 min at 4°C. Then, the cells were stained with 2 µL of FITC‐conjugated antimouse CD48 antibody (BioLegend, San Diego, CA, USA), 2 µL of PE‐conjugated antimouse c‐kit antibody (BD Biosciences), 2 µL of PE‐Cy7‐conjugated antimouse Sca‐1 antibody (BD Biosciences), and 10 µL of APC‐conjugated antilineage antibody (BD Biosciences). After incubation for 60 min at 4°C, the cells were washed twice. After adding 10 µL of 7‐amino‐actinomycin D (BD Biosciences) to remove dead cells, the cells were analyzed using FACS Canto II (BD Biosciences). HSCs (CD48^−^Lin^−^Sca‐1^+^c‐Kit^+^) in the PB and spleen were analyzed. Data were analyzed using FlowJo software (BD Biosciences).

### 2.4 Patient Samples

We retrospectively included 47 patients diagnosed with polycythemia vera (PV), essential thrombocythemia (ET), pre‐PMF, or PMF at National Defense Medical College Hospital between 2017 and 2018 based on the WHO classification [1]. Forty‐seven patients were classified into three groups: MF‐0 (*n* = 21), MF‐1 (*n* = 17), and MF‐2 (*n* = 9) according to the degree of BM fibrosis (Table 1). Patients who had been previously treated with ruxolitinib were excluded to avoid potential confounding effects of the drug. A BM biopsy was performed simultaneously with the collection of a PB sample. The degree of BM fibrosis was assessed by pathologists according to the European consensus criteria [2]. Furthermore, the *JAK2*V617F mutation was assessed using genomic DNA isolated from the PB of all patients, as described in a previous study [20].

**TABLE 1:** Characteristics of patients with myeloproliferative neoplasms who presented with MF‐0, MF‐1, and MF‐2.

	Patients with MF‐0	Patients with MF‐1	Patients with MF‐2
Number of patients	21	17	9
Men/women	8/13	8/9	4/5
Age (years), mean ± SD	63.0 ± 16.6	69.2 ± 12.8	65.7 ± 14.0
Genetic mutation			
*JAK2*V617F	8	7	4
Primary disease			
PV	10	12	1
ET	11	4	6
PMF	0	0	2
Pre‐PMF	0	1	0
Medication			
Hydroxyurea	7	5	3
Anagrelide	0	0	1
Ruxolitinib	0	0	0
Interferon	0	0	0

Abbreviations: ET, essential thrombocythemia; MF, myelofibrosis; MPN, myeloproliferative neoplasm; PMF, primary myelofibrosis; pre‐PMF, prefibrotic primary myelofibrosis; PV, polycythemia vera; SD, standard deviation.

### 2.5 Statistical Analysis

Data are expressed as mean ± standard error of the mean. Differences between two experimental groups were assessed using Mann–Whitney *U* test, and differences among more than two experimental groups were evaluated using one‐way analysis of variance with post‐hoc Steel–Dwass test. All statistical analyses were conducted with a significance level of *α* = 0.05 (*p* < 0.05). GraphPad Prism 7.0 (GraphPad Software, La Jolla, CA, USA) and JMP software (version 14; SAS Institute Inc., Cary, NC) were used for data visualization and statistical analysis.

## 3 Results

### 3.1 Changes in PMF‐Related Events and HSC Dynamics in a Drug‐Induced Murine MF Model Using Romiplostim

**FIGURE 5:**
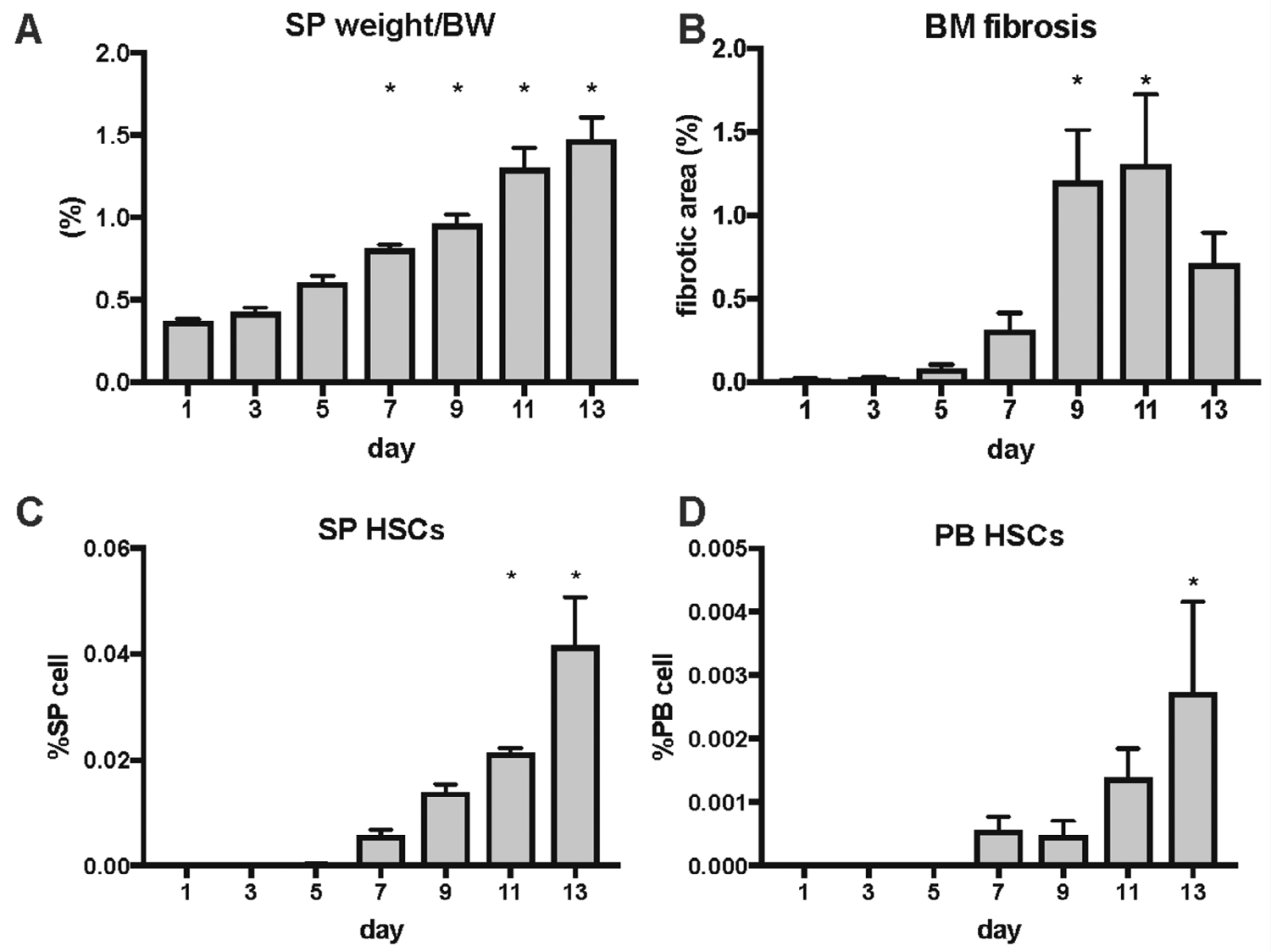
Sequential changes in MF‐related events in the Rom MF model. (A) Sequential change in spleen weight over time (*n* = 4 per day). (B) Quantified fibrotic area using a deep learning method (*n* = 4 per day). (C, D) The percentage of hematopoietic stem cells in the spleen and peripheral blood was analyzed using fluorescence‐activated cell sorting (*n* = 4 per day). *p < 0.05 indicates a significant difference compared to Day 1. BM, bone marrow; HSCs, hematopoietic stem cells; MF, myelofibrosis; Rom, romiplostim; SP, spleen.

The mean percentage of spleen weight relative to body weight, evaluated on alternate days from Day 1 to 13, was 0.4%, 0.4%, 0.6%, 0.8%, 1.0%, 1.3%, and 1.5%, respectively (Figure 5A). The percentage of spleen weight relative to body weight in the Rom MF model mice significantly increased compared with that in the control mice on Day 7 following the administration of Rom and gradually worsened (*p* < 0.05). The mean fibrotic areas measured on alternate days from Day 1 to 13 were 0.015%, 0.021%, 0.081%, 0.31%, 1.20%, 1.31%, and 0.71%, respectively (Figure 5B). BM fibrosis peaked on Days 9 and 11 following Rom administration (*p* < 0.05). The mean percentages of HSCs in the spleen cells, measured on alternate days from Day 1 to 13, were 0.00%, 0.00%, 0.00%, 0.005%, 0.014%, 0.021%, and 0.042%, respectively (Figure 5C). The mean percentage of HSCs in the PB cells, measured on alternate days from Day 1 to 13 following Rom administration, was 0.00%, 0.00%, 0.00%, 0.0006%, 0.0005%, 0.0014%, and 0.0027%, respectively (Figure 5D). HSCs in the spleen and PB increased on Days 11 and 13, respectively, after Rom administration. Splenomegaly occurred, followed by BM fibrosis and an increase in HSCs in spleen and PB.

### 3.2 Changes in PMF‐Related Events and HSC Dynamics in *Jak*2V617F Gene–Transformed Mice

**FIGURE 6:**
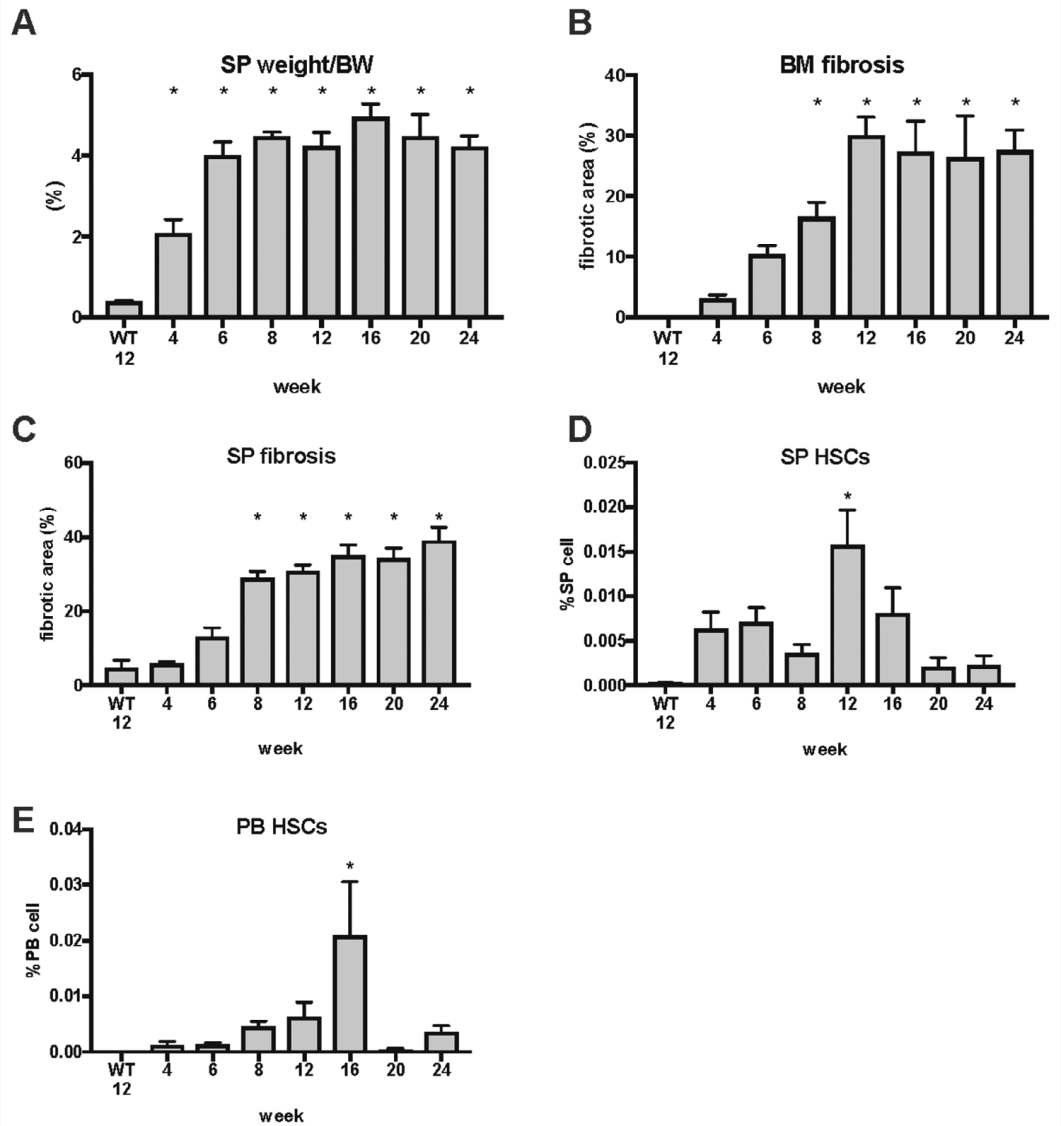
Sequential changes in MF‐related events in Jak2V617F transgenic mice. (A) Changes in spleen weight over time (*n* = 4–6 per age group). (B) Quantified bone marrow fibrotic area using a deep learning method (*n* = 4–6 per age group). (C) Quantified spleen fibrotic area using a deep learning method (*n* = 4–6 per age group). (D, E) The percentage of hematopoietic stem cells in the spleen and peripheral blood was analyzed using fluorescence‐activated cell sorting (*n* = 4–6 per age group). *p < 0.05 indicates a significant difference from WT 12. BM, bone marrow; HSCs, hematopoietic stem cells; MF, myelofibrosis; SP, spleen; WT 12, wild‐type mice aged 12 weeks.

*Jak2*V617F TG mice were euthanized at the ages of 4, 6, 8, 12, 16, 20, and 24 weeks (*n* = 4–6 per age group). The mean percentages of spleen weight to body weight at the ages of 4, 6, 8, 12, 16, 20, and 24 weeks were 2.10%, 4.00%, 4.47%, 4.23%, 4.96%, 4.48%, and 4.23%, respectively. In contrast, the mean percentage of spleen weight to body weight in 12‐week‐old wild‐type mice was 0.39% (Figure 6A). The mean BM fibrotic areas at 4, 6, 8, 12, 16, 20, and 24 weeks of age were 3.06%, 10.43%, 16.58%, 30.02%, 27.37%, 26.43%, and 27.56%, respectively. In contrast, the mean BM fibrotic area in 12‐week‐old wild‐type mice was only 0.01% (Figure 6B). The mean fibrotic areas of the spleen at 4, 6, 8, 12, 16, 20, and 24 weeks of age were 5.87%, 13.10%, 29.05%, 30.82%, 35.10%, 34.39%, and 39.20%, respectively. In comparison, the mean fibrotic area of the spleen in 12‐week‐old wild‐type mice was 4.70% (Figure 6C). The mean percentages of HSCs in the spleen cells measured at 4, 6, 8, 12, 16, 20, and 24 weeks of age were 0.0063%, 0.0071%, 0.0036%, 0.0158%, 0.0080%, 0.0021%, and 0.0023%, respectively. In contrast, the mean percentage of HSCs in the spleen cells of 12‐week‐old wild‐type mice was 0.00% (Figure 6D). The mean percentages of HSCs in the PB cells measured at 4, 6, 8, 12, 16, 20, and 24 weeks of age were 0.0013%, 0.0013%, 0.0046%, 0.0063%, 0.0208%, 0.0003%, and 0.0036%, respectively. In contrast, the mean percentage of HSCs in the PB cells of 12‐week‐old wild‐type mice was 0.00% (Figure 6E). Compared with 12‐week‐old wild‐type mice, *Jak2*V617F TG mice showed significant splenomegaly at 4 weeks of age, significant BM fibrosis and spleen fibrosis at 8 weeks of age, and significant increases in HSCs in the spleen and PB, similar to the Rom MF model mice. Notably, splenomegaly preceded BM fibrosis. The increase in megakaryocyte counts peaked at 4 weeks of age, indicating that EMH was active during this period.

### 3.3 Application of the Deep Learning‐Based Method for Quantifying Fibrosis in the Samples of Patients with MPN

**FIGURE 7:**
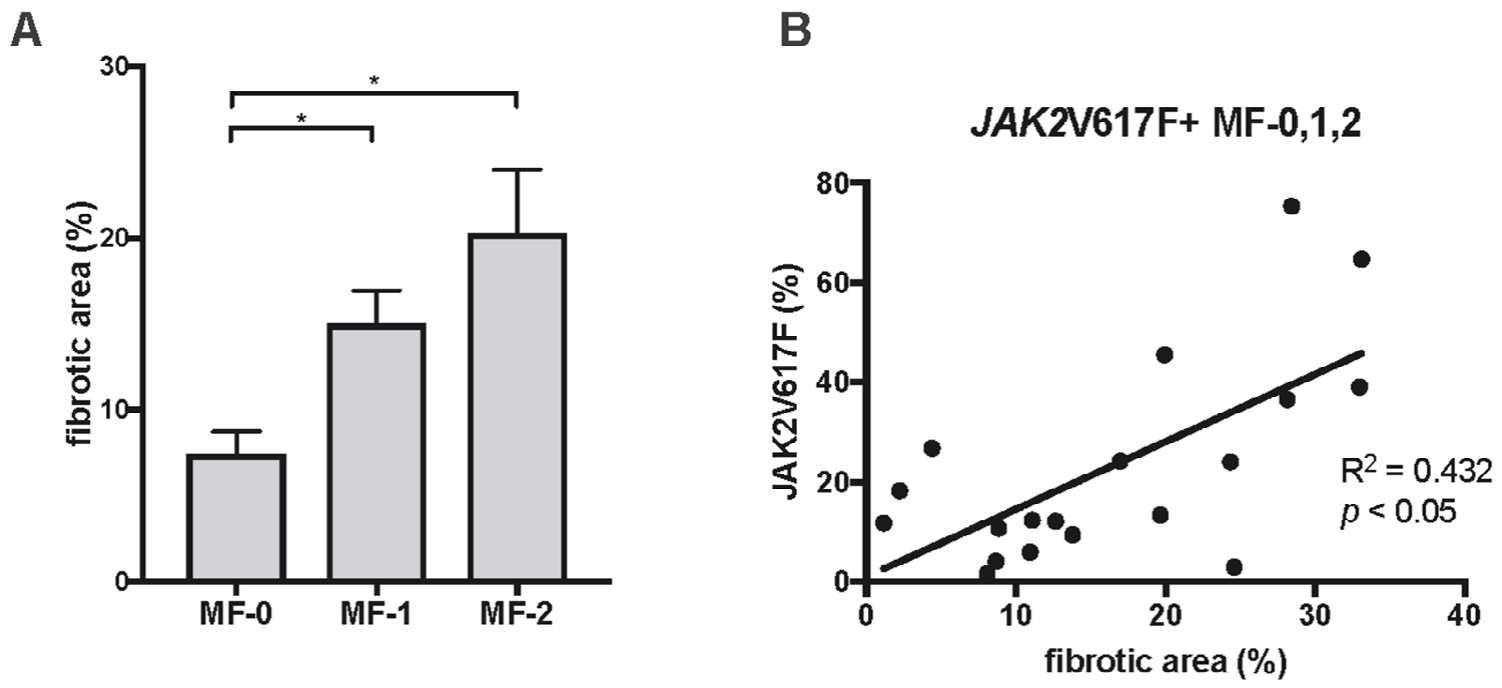
Utility of the deep learning‐based fibrosis quantification method and the correlation between fibrotic area and JAK2V617F allele burden in patients with MPNs. (A) Comparison of the fibrotic area in patients with MPNs, categorized according to MF‐0, MF‐1, and MF‐2 (*n* = 21, 17, and 9). (B) Correlation between fibrotic area and JAK2V617F allele burden in patients with JAK2V617F mutation‐positive MPNs presenting with MF‐0–2 (*n* = 19). *p < 0.05. MF, myelofibrosis; MPN, myeloproliferative neoplasm.

In 47 patients with MPNs, BM fibrosis was assessed using a deep learning‐based fibrosis quantification method (Table 1). Since this quantitative method cannot assess collagen fibers, patients with MPNs presenting as MF‐3 were not assessed for this parameter. The mean fibrotic areas in the BM of patients with MPNs who presented with MF‐0, MF‐1, and MF‐2 were 7.41%, 15.00%, and 20.30%, respectively (Figure 7A). The fibrotic areas in patients with MPNs presenting with MF‐1 and MF‐2 were significantly larger than those in patients presenting with MF‐0 (*p* < 0.05). However, no significant difference was observed between patients with MPNs presenting as MF‐1 and those presenting as MF‐2 (*p* = 0.2543). The fibrotic area in patients with MPNs was significantly correlated with the *JAK2*V617F allele burden (*R* ^2^ = 0.432, *p *< 0.05; Figure 7B). This new quantitative method enabled stratification down to MF‐2, and the fibrotic area correlated with the number of *JAK2*V617F mutations.

## 4 Discussion

We observed time‐dependent linear progression of BM fibrosis using our deep learning‐based method in two murine MF models: Rom‐induced MF mice and *Jak2*V617F TG mice. Several deep learning methods have recently been developed to diagnose hematological malignancies [21, 22, 23]. Most reports assessing fibrosis with deep learning have focused on the liver and lungs [24, 25], with none of the studies concentrating on the BM. Here, we used deep learning to assess spleen fibrosis, which had not been accurately evaluated before. Our deep learning‐based quantification of BM and spleen fibrosis described more detailed changes compared with commonly used qualitative evaluations and enabled us to assess differences in the severity of BM fibrosis in low‐quantity human samples. The analysis time was only a few minutes per sample after importing the images into HALO AI, suggesting easy integration into the clinical field.

BM fibrosis, EMH, and splenomegaly are the major clinical symptoms of PMF. Although their sequence of occurrence has not been completely elucidated, our method enabled us to sequentially evaluate the relationships among them. The association between certain events related to PMF has been reported. The migration of HSCs from the BM niches and the emergence of EMH are associated with *JAK2* activation and several cytokines, including CXC motif chemokine ligand 12 (CXCL12) and CXC chemokine receptor Type 4 (CXCR4) [26, 27, 28, 29]. *JAK2* activation, a driver mutation, spontaneously activates the CXCL12/CXCR4 pathway and promotes EMH, leading to progressive splenomegaly [30].

In the Rom MF model, splenomegaly developed before BM fibrosis, and HSCs increased in spleen and PB. BM fibrosis peaked on Day 11 and subsided by Day 13. Thus, BM fibrosis in the Rom MF model spontaneously resolved when Rom was discontinued.

*Jak2*V617F TG mice showed significant splenomegaly and an increased fraction of megakaryocytes in the spleen at 4 weeks of age, as well as significant BM and spleen fibrosis at 8 weeks of age compared with wild‐type mice. Although no significant increase in HSCs was observed in the spleen at 4 weeks of age, the significant increase in megakaryocytes suggested that EMH occurred in the spleen at this time.

Interestingly, our study demonstrated that in both the Rom MF model, an acute inflammation model, and the *Jak2*V617F TG mice, a chronic neoplastic disease model—two models that differ fundamentally in mechanism and nature—splenomegaly and EMH in the spleen preceded the development of MF. In a report describing EMH in the spleen, Tlx1‐expressing mesenchymal progenitor‐like cells were associated with niche formation and EMH regulation in the spleen [31]. The connection between Tlx1‐expressing mesenchymal progenitor‐like cells and early splenomegaly in murine MF models requires further investigation. We considered analyzing HSCs in the BM, but collecting BM cells proved difficult due to the development of fibrosis.

Our analysis of patients’ silver‐stained specimens using quantitative methods showed that the size of the fibrotic area tended to increase with an increase in MF grades. In *JAK2*V617F mutation‐positive patients with MPNs, a positive correlation was observed between the *JAK2*V617F allele burden and fibrotic area. A high *JAK2*V617F allele burden is a risk factor for thrombosis and progression to secondary MF in patients with PV and ET [32, 33, 34]. The fibrotic area may also be a prognostic factor. However, negative clinical data have been obtained regarding the association between treatment‐induced changes in BM fibrosis and efficacy outcomes [11]. If changes in BM fibrosis can be assessed using the novel deep learning‐based method for quantifying fibrosis, the relationship to efficacy outcomes may become clearer. However, a key limitation of this method is its difficulty in assessing MF‐3 cases, where extensive bone sclerosis and the scarcity of hematopoietic tissue hinder reliable evaluation.

## 5 Conclusion

We developed a novel deep learning‐based method for quantifying fibrosis and analyzed PMF‐related events (splenomegaly, BM fibrosis, spleen fibrosis, and EMH) and HSC dynamics in murine MF models. Deep learning enables the monitoring of changes in BM and spleen fibrosis over time, which was challenging in the past and is expected to significantly advance this field. Our results demonstrated that splenomegaly precedes BM fibrosis in vivo. Nevertheless, further research is needed to identify factors contributing to the development of splenomegaly.

## Funding

This work was partly supported by the Japan Society for the Promotion of Science Grants‐in‐Aid for Scientific Research No. 18K08344.

## Ethics Statement

All animal procedures and experiments were approved by the Animal Experiment Ethics Committee of the National Defense Medical College (approval number: 19056) and performed in accordance with the Animal Research: Reporting of In Vivo Experiments guidelines and the American Veterinary Medical Association Guidelines for the Euthanasia of Animals.

## Consent

This study involving human subjects was approved by the Institutional Review Board of the National Defense Medical College (approval number: 2877) and was conducted in accordance with the Declaration of Helsinki. All patients provided written informed consent.

## Conflicts of Interest

The authors declare no conflicts of interest.

## Data Availability

The datasets generated during and/or analyzed during the current study are available from the corresponding author on reasonable request.
